# Implementation of an Online Auditory Attention Detection Model with Electroencephalography in a Dichotomous Listening Experiment

**DOI:** 10.3390/s21020531

**Published:** 2021-01-13

**Authors:** Seung-Cheol Baek, Jae Ho Chung, Yoonseob Lim

**Affiliations:** 1 Center for Intelligent & Interactive Robotics, Artificial Intelligence and Robot Institute, Korea Institute of Science and Technology, Seoul 02792, Korea; ggachi121@kist.re.kr; 2 Department of Otolaryngology-Head and Neck Surgery, College of Medicine, Hanyang University, Seoul 04763, Korea; 3 Department of HY-KIST Bio-convergence, Hanyang University, Seoul 04763, Korea; 4 Research Center for Diagnosis, Treatment and Care System of Dementia, Korea Institute of Science and Technology, Seoul 02792, Korea

**Keywords:** online auditory attention detection, electroencephalography, linear decoder model, sliding window, dichotomous listening

## Abstract

Auditory attention detection (AAD) is the tracking of a sound source to which a listener is attending based on neural signals. Despite expectation for the applicability of AAD in real-life, most AAD research has been conducted on recorded electroencephalograms (EEGs), which is far from online implementation. In the present study, we attempted to propose an online AAD model and to implement it on a streaming EEG. The proposed model was devised by introducing a sliding window into the linear decoder model and was simulated using two datasets obtained from separate experiments to evaluate the feasibility. After simulation, the online model was constructed and evaluated based on the streaming EEG of an individual, acquired during a dichotomous listening experiment. Our model was able to detect the transient direction of a participant’s attention on the order of one second during the experiment and showed up to 70% average detection accuracy. We expect that the proposed online model could be applied to develop adaptive hearing aids or neurofeedback training for auditory attention and speech perception.

## 1. Introduction

One of the astounding abilities of humans is to extract and recognize a sound even in a noisy situation. Since it was described for the first time in the mid-1950s and formalized as the cocktail party effect [1], this remarkable ability has been analyzed by a large number of researchers. It remains uncertain how humans can separate a particular sound object from other sound signals and related neural mechanisms. However, collective findings from several decades suggest that selective auditory attention plays a central role in this ability. Selective auditory attention is known to modulate the neural representation of sounds [2,3]. In other words, an attended sound is represented as relatively strong at the neural level, while an ignored one is weak. This implies that selective auditory attention can influence the degree of perception of each sound [4,5].

These findings allow the tracking of a sound attended by a listener based on neural response in a complex auditory scene. This process is called auditory attention detection (AAD) and is expected to be applicable in various areas of life. A potential application is adaptive hearing aids to selectively amplify the magnitude of attended speech and dampen that not of interest [6,7].

In this sense, electroencephalography (EEG) is favorable in AAD research because it is inexpensive, light-weight, and easy to access [8,9]. Various AAD methods have been proposed using EEG. In earlier attempts, attended and ignored sounds could be discriminated by comparing the amplitude or latency of event-related potential (ERP) components elicited by auditory stimuli, such as P300 [10,11]. However, this method was inefficient because, in order to obtain ERP response to a single sound, it was required to average neural responses time-locked to the same sound repetitively given to subjects. In addition, this method was inappropriate for sound stimuli whose acoustical property changed dynamically across time as in natural speech.

Some researchers have tried to detect auditory attention based on features extracted from EEG, including entropy [12], auditory steady-state responses [13], degree of lateralization of alpha power [13,14], and effective connectivity pattern between electrodes [9]. AAD methods based on the degree of lateralization of alpha power or auditory steady-state response showed relatively low accuracy, and EEG data from multi-trials were needed to measure these features, which was one of the biggest obstacles for online implementation. Additionally, calculating the features like entropy or effective connectivity is computationally intensive.

One of the most effective and widely used methods for AAD is construction of mapping functions between speech envelopes in the low-frequency range (i.e., 2–8 Hz) and neural responses in a forward (i.e., encoding) or backward (i.e., decoding or reconstruction) manner using a linear or non-linear model [15,16,17]. A rationale for this is that the low-frequency component of speech is represented as neural entrainment in the cortex and can be modulated by selective attention of the listener [5,18,19]. In this sense, through this approach, it was possible to partly investigate the neural representation of speech and to detect one’s auditory attention based on single-trial EEG data, but a relatively longer segment of EEG and corresponding speech envelope were needed for a single detection. Non-linear models such as neural networks showed better detection performance compared to linear models [17,20]. Nonetheless, a linear decoder model was more widely investigated due to reasonable detection accuracy and faster model training compared to non-linear models [15,21].

To apply AAD methods to real life, efforts have been made to reduce the length of data needed for detection [16,21] or the number of electrodes to lessen computational cost [22]. However, none of these studies has attempted to implement AAD with streaming EEG data. Even in a recent study that proposed an online AAD method working, the method was simulated on a recorded EEG [23]. To our knowledge, only a few studies have conducted research on online AAD while streaming EEG data. Haghighi et al. trained a classifier on cross-correlation features between speech and neural signals and adjusted the gain of both attended and ignored speech based on online detection results [6]. Zink et al. applied their model to feedback training, in which the size or color of a circle implicitly indicating the attention level of the listener is provided as online feedback [8]. However, the temporal resolution of both online AAD models was on the order of 10 s (i.e., sparse detection results), and it has not been determined whether the model can capture instantaneous change in the direction of listener’s attention.

In the present study, we introduce a simple online AAD method that outputs frequent detection results to track the transient direction of a listener’s attention. The proposed decoder model is based on the linear decoder model that has been widely used for auditory attention detection from EEG recordings [21]. The feasibility of the proposed method was tested with offline simulation and a real online experiment in the lab. We expect that our model can be built and tested online based on the streamed EEG and will show acceptable detection accuracy. In addition, the model will capture the change in direction of a listener’s attention within a few seconds.

## 2. Materials and Methods

### 2.1. Online Auditory Attention Detection Model

#### 2.1.1. The Linear Decoder Model

A linear decoder model D(τ,n) can be formalized as a function that maps the EEG signal of a channel n, R(t,n) to the envelope of the sound stimulus S(t) at time t as below:

(1) $$\hat{S}\left(t\right)=\;\sum_{n}\sum_{\tau }D\left(\tau ,n\right)R\left(t\;-\;\tau ,n\right),$$

where $\hat{S}\left(t\right)$ denotes the estimated speech envelop at time t, and τ is the time-lag reflecting the latency of EEG signals in response to speech.

The decoder model D(τ,n) can be estimated by solving the formula as follows:

(2) $$D={\left(RR^{T}+\lambda I\right)}^{-1}RS^{T},$$

where λ is an L2 regularization parameter to prevent overfitting.

#### 2.1.2. Online Decoder Model Construction

**Figure 1:**
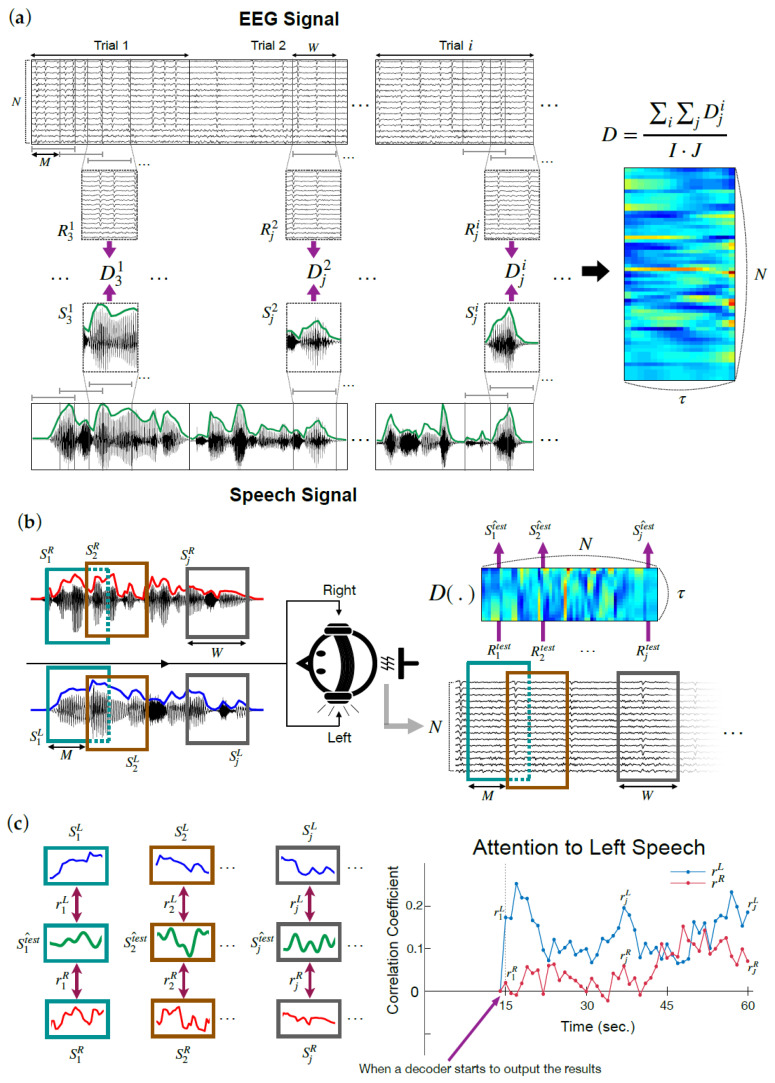
Online decoder model. (**a**) Construction of an online decoder model. Online decoder model (D) is the average of the individual decoders ($D_{j}^{i}$) estimated with snippets of EEG signal ($R_{j}^{i}$) and corresponding speech signal ($S_{j}^{i}$). i and j are the index of trial and snippet, respectively. (**b**) Implementation of the online decoder model in a dichotic listening scenario. (**c**) Detection of the direction of auditory attention based on the correlation between reconstructed and actual speech envelopes. An example of online detection results is plotted on the right.

To develop a new online AAD model, we applied a sliding window that allows an overlap to the conventional decoding model (Figure 1a). The proposed online AAD model was built by averaging all decoder weights estimated from each EEG data snippet, acquired by a sliding window at a given interval. Construction of the online decoder model is defined as follows.

Similar to the existing decoder model, a decoder $D_{j}\left(\tau ,n\right)$ that maps a snippet of EEG signal of a channel n, $R_{j}\left(t_{j},n\right)$ to a corresponding snippet of speech $S_{j}\left(t_{j}\right)$ at time $t_{j}$ can be formalized as:

(3) $${\hat{S}}_{j}\left(t_{j}\right)=\;\sum_{n}\sum_{\tau }D_{j}\left(\tau ,n\right)R_{j}\left(t_{j}\;-\;\tau ,n\right),$$

where ${\hat{S}}_{j}\left(t_{j}\right)$ is the estimated envelope of a speech snippet at time $t_{j}$, and τ is the time-lag parameter. Given a length of speech corresponding to a single trial T, a snippet of speech $S_{j}\left(t_{j}\right)$ at time $t_{j}$ is extracted by an overlapping sliding window, where the subscript j denotes a snippet from the j-th window; and $t_{j},$ a subset of t=1, ⋯, T, spans from (j − 1)·M+1 to (j − 1)·M+W. Here, W denotes the size of a window, and M represents the window hopping size of sliding when a window moves. Thereby, J, which is the number of snippets belonging to each trial, is based on W and M as follows: $J=⎣\frac{T\;-\;W+1}{M}⎦$, where the open square brackets denote a floor function. If window size W is equal to trial length T, and M is 0, then Equations (1) and (3) are the same. The snippet-wise decoder model $D_{j}$ can be estimated in the same way as in Equation (4):

(4) $$D_{j}={\left(R_{j}R_{j}^{T}+\lambda I\right)}^{-1}R_{j}S_{j}^{T},$$

where λ is likewise an L2 regularization parameter. Finally, an online AAD model is built by averaging all $D_{j}$ over every trial held for the model construction as in Equation (5):

(5) $$D=\;\frac{\sum_{i}\sum_{j}D_{j}^{i}}{I\cdot J},$$

where superscript i=1, ⋯, I refers to the i-th trial used for model construction.

Figure 1b illustrates the online process of decoding auditory attention in a dichotic listening condition. While two different speech sounds are delivered to each ear of the listener, a snippet of EEG signal $R_{j}^{test}$ is extracted and fed to the decoder model to reconstruct a speech envelope ${\hat{S}}_{j}^{test}$. At the same time, snippets of speech envelopes $S_{j}^{L}$ and $S_{j}^{R}$ are extracted and compared to the reconstructed speech envelope based on Pearson correlation (Figure 1c). If the correlation coefficient between the reconstructed and actual speech delivered to the left $r_{j}^{L}$ is larger than that between the reconstructed and speech to the right $r_{j}^{R}$, the model indicates that the listener attends to the left speech at time point (j − 1)·M+W, and vice versa. As an example, the detection results of a single trial from our experiment are plotted on the right panel of Figure 1c.

#### 2.1.3. Direction-Biased Model

In addition to an online AAD model introduced in Section 2.1.2, we propose a slightly modified version of the model which we call a direction-biased model. A direction-biased model is basically the same as the online AAD model except that it is constructed only on trials in which a listener attended to the same side. Since in this paper a dichotic listening condition is assumed, two types of direction-biased model can be made: a left- and right-biased model. These two types of a direction-biased model function as a counterpart on a single online AAD model.

A rationale for the proposal of a direction-biased model is the reports from previous studies which alluded that AAD models can take advantage of the spatial bias of auditory attention. According to a recent report from Das and colleagues, the decoder model trained and evaluated in the same direction performed better than that trained and evaluated in both directions, or trained in one direction and evaluated in another [24]. Moreover, Geravanchizadeh et al. reported that effective connectivity between scalp electrodes showed different patterns when the listener attended to left, or right speech [9]. These results imply different underlying networks involved in the processing of the sides of speech, and idiosyncratic features when processing each side can be captured during training AAD models and can contribute more to detect the direction of the listener’s attention to the corresponding side. As such, to determine whether our online AAD model benefits from the spatial effect, we additionally constructed a pair of direction-biased decoders (henceforth, biased models) and tested whether biased models would outperform a single-decoder model (hereafter, a single model).

For a fair comparison, each direction-biased model was constructed on half of the model construction trials I in which the listener attended to the same direction. In other words, a left-biased decoder model was built on the model construction trials in which the listener attended only to the left side, and a right-biased model was built on the trials in which the listener attended only to the right side. By doing so, the same number of the model construction trials I was used for biased models and a single model. In testing time, each biased model was evaluated only when the direction of the listener’s attention in each test trial was the same as in the trials used for its construction. That is, a biased model constructed on the left trials was evaluated on the left test trials, while a biased model constructed on the right trials was evaluated on the right test trials.

### 2.2. Online AAD Simulation

#### 2.2.1. Dataset

Two datasets were used to simulate the proposed online AAD method. These datasets consisted of preprocessed speech envelopes and EEG data, which were obtained from two dichotic listening experiments in our previous study [25].

##### Direction-Fixed Dataset

The direction-fixed dataset was obtained with a fixed direction of attention throughout the task. As in the previous AAD study [21], speech stimuli were constructed based on two stories, “*Journey to the center of the earth*” (henceforth, *Journey*) and “*Twenty thousand leagues under the sea*” (henceforth, *Twenty*) by Jules Verne. The Korean translations of the stories were used and were recorded in a male voice (sampled at 44.1 Hz). After recording, each story was divided into 30 1-min segments, with all pauses truncated to less than 500 ms. In addition, all speech segments were normalized to the same root square mean (RMS) level (0.8).

The experiment consisted of 30 trials, each comprised of a speech segment from one of the stories. *Twenty* was delivered to the left ear via air-conduction earphones (ER2, Etymotic Research, IL, USA), while *Journey* was sent to the right. All participants listened to both stories and had to attend to one of the stories throughout the task.

The speech envelope data of the direction-fixed dataset were obtained as follows. First, the Hilbert transform was applied to each segment of speech, and the raw speech envelope was produced by calculating the power of the analytic signal. This raw speech envelope was subsequently downsampled to 64 Hz and z-scored. This resulted in a total of 60 preprocessed speech envelopes corresponding to the 30 trials of speech stimuli (30 trials for two stories).

The EEG data were obtained from 10 participants using a 64-electrode system with Neuroscan SynAmps RT (64-channel Quik-Cap, Compumedics, Victoria, Australia). During recording, raw EEG data were referenced to the voltage from an electrode located between the electrode sites Cz and CPz and were sampled at a rate of 1000 Hz. After acquisition, a high-pass filter with a 0.5 cut-off frequency and a notch filter at 60 and 120 Hz were applied to the raw EEG data. The filtered data were re-referenced to a common average reference excluding vertical and horizontal electrooculograms (V/HEOG). Next, a bandpass filter was applied to extract the 2–8 Hz frequency band of the EEG signal [21]. Finally, the EEG data were segmented corresponding to the onset and offset of speech stimuli per trial, downsampled to 64 Hz, and z-scored. This resulted in a total of 30 preprocessed EEG segments corresponding to the 30 trials.

##### Direction-Switching Dataset

The direction-switching dataset was obtained from the experiment in which the direction of attention was set randomly before each trial. In the experiment, two different speech segments were dichotically presented to a participant for each trial, as in the experiment of the direction-fixed dataset. However, a participant had to change their attention across trials because the direction that a participant had to attend to was determined randomly for each trial. The direction of attention was cued before the onset of each trial, and the probability of attending to the left or right was equal at 50%. A total of 10 participants took part in the experiment.

For a set of speech stimuli used in the task, 60 speeches were excerpted from a Korean listening comprehension test sample for high school students, which were recorded in a female voice and sampled at a rate of 44.1 kHz. These samples were segmented to a length of 1 min, while the silences within these segments were truncated to less than 500 ms. Additionally, the RMS intensity of every segment was normalized to the same level (0.8). Unlike the speech stimuli used in the direction-fixed dataset, each speech segment was independent in a contextual sense because each had its own topic.

The experiment also consisted of 30 trials, each of which comprised two speech segments delivered to each ear via air-conduction earphones. Not only was the direction of attention randomized, but the order of presentation of stimuli was randomly decided. No speech segment was presented twice. The speech envelope data and the EEG data were obtained by applying the same preprocessing procedure as in the direction-fixed dataset. As a result of the preprocessing, 60 1-min-long speech envelopes and 30 preprocessed EEG segments were involved in the 30 trials.

#### 2.2.2. Parameter Selection

In our online decoder model, three parameters were newly introduced: window size W, slide hopping size M, and total number of trials used in model construction I. To determine the effect of each parameter on detection accuracy and to select the values of these parameters, we searched for the optimal combination of these parameters in their joint parameter space. Online decoder models were built and evaluated using the two datasets mentioned in Section 2.2.1 for every combination of parameters. The values of the parameters were selected based on accuracy. The L2 regularization parameter λ and the time-lag parameter τ were fixed to 10 and 250 ms, respectively, based on pilot simulation results and a previous study (applied equally to the latter simulation and the online AAD experiment) [16,21].

A set of candidates for each parameter was set as follows. In terms of W, O’Sullivan et al. argued for a value between 15 and 20 s to allow the decoder model robustly to detect the direction of attention and react sensitively enough to changes in direction of attention [26]. On the other hand, Wong et al. recommended W between 3 and 5 s to achieve the maximum bit rate [16]. Thus, a set of values {5, 10, 15, 20} s was chosen for the possible W. For M, we searched for the best size among {1, 3, 5, 7, 9} s. We did not investigate cases of M at 10 or more based on the fact that an online AAD model proposed by Zink et al. operated with time resolution of 10 s [8]. Lastly, a set of possible candidates {1, 5, 10, 15} was selected for the first I trials used in model construction. Considering that the usual EEG experiment was designed to require no more than 40–50 min to prevent subjects’ exhaustion [27], it would be difficult to perform more than 15 trials (i.e., 15 min of speech) for model construction.

#### 2.2.3. Smoothing of Correlation Coefficients

Correlation coefficients produced by the online decoder model were smoothed within each trial with a moving average filter by averaging the previous k samples. If the number of samples was less than k − 1, the smoothed value was calculated based on the average of all samples including the correlation coefficient itself. The simulation was performed for the cases with moving average filter width of 1, 3, 5, and 7. A k equal to one is equivalent to use of no filter.

### 2.3. Online AAD Experiment

In addition to the online AAD simulation, we attempted to test our online AAD model by streaming EEG signals online. In this section, experimental settings are explained in detail.

#### 2.3.1. Subjects

Six participants (1 female) aged 24 to 26 years were recruited. All were native Korean speakers and self-reported that they were right-handed with normal hearing and no history of neurological disorder. Participants provided written informed consent and were reimbursed for participation. All experimental procedures followed the ethical standards of the Declaration of Helsinki and were approved by the Institutional Review Boards of Korea Institute of Science and Technology and Seoul National University Hospital (IRB code: 2019-044/2020-017 and H-1706-137-861, respectively).

#### 2.3.2. Stimuli

The same speech stimuli as mentioned in the explanation of the direction-fixed dataset were used in this experiment: excerpts from *Journey* and *Twenty*, read by two male speakers. For each story, 30 1-min segments were derived. There was no pause longer than 0.5 s, and the RMS level of each segment was normalized to 0.8. As a result, a total of 60 speech segments were used as the experimental stimuli for each participant.

#### 2.3.3. Experimental Procedure

**Figure 2:**
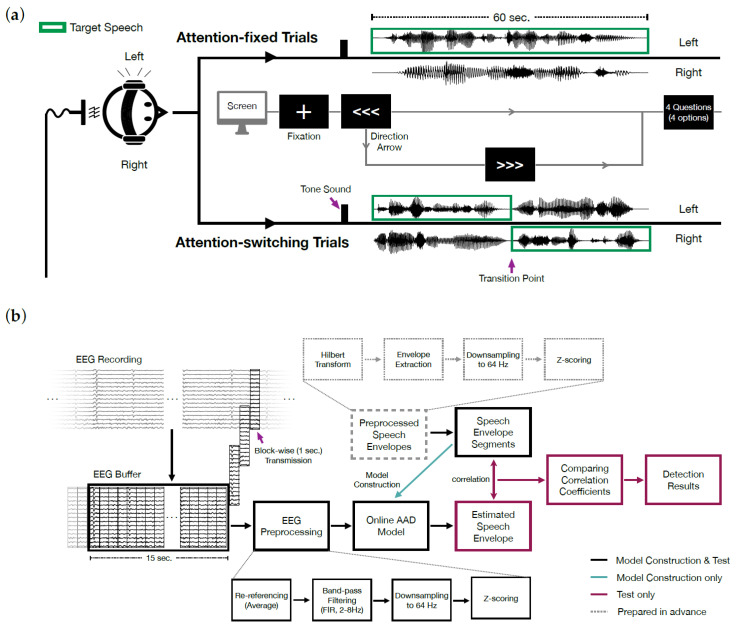
Online auditory attention detection (AAD) experiment. (**a**) An illustration of the experimental procedure. (**b**) Online data processing pipeline for model construction and testing.

The experiment was conducted in a dimly lit and soundproof chamber with participants sitting in a comfortable chair. Instructions and visual cues were presented via a monitor, and the speech stimuli were delivered to the participants through air-conduction earphones. During the experiment, participants were asked to attend only to the story *Journey* (i.e., target speech, Figure 2a) presented in either the left or right ear, while ignoring the story *Twenty* presented on the other side. Both stories unfolded over 30 trials through a 1-min long speech segment per trial for each of the six participants.

Experimental trials consisted of two types: attention-fixed trials (26 trials) and attention-switching trials (four trials). The first 14 attention-fixed trials were used to build the online AAD model, while the rest of the trials (12 attention-fixed trials and 4 attention-switching trials) were for model evaluation. In the attention-fixed trials, target speech was presented only in one direction. However, in attention-switching trials, the target speech changed sides at a transition point. Hence, participants had to change their direction of attention within each attention-switching trial. Transitions occurred near the middle point of the speech sample.

Figure 2a shows the schematic flow of the experiment procedure. When each trial began, a fixation cross was presented on the screen for 500 ms. Then, two types of cues indicating the direction of target speech (i.e., the side from which *Journey* was presented) were simultaneously presented: a tone sound and a directional arrow. A tone sound was presented for 2 s only to the side on which the target speech was to be delivered, simultaneous with an arrow pointing in the direction of attention. After 3 s from the onset of the tone sound, two speech segments from each story were presented to each ear for 60 s. In the attention-fixed trials, the arrow remained on the screen until the end of speech stimuli. However, in the attention-switching trials, one of the wedged shapes in the arrow was eliminated every second from 3 s before the transition point. At the transition point, an intact direction arrow was presented to the opposite side to signal the change of direction. Participants had to attend their gaze on the center of the screen during each trial.

To confirm that the participants attended only to the target speech and ignored the other, after each trial, participants were given four questions with four options. Two were about the target speech (*Journey*), and the other two were about the ignored speech (*Twenty*). Moreover, since the length of each segment was 1-min, half of the questions were about the first half of the speech segments, and the other half were about the second half. There was no time limit to answering the questions. After participants responded to four questions, the next trial began.

During the experiment, both types of online decoder models, a single model and biased models, were constructed for each participant during the first 14 trials (14 attention-fixed trials) based on the streamed EEG. While a single model was built on all 14 trials, each direction-biased model was constructed on seven trials in which the target speeches were presented in the same direction (seven left- and right-attention trials for each biased model).

After construction, the trained decoder models were used to detect the direction of participants’ attention during 16 test trials. Among these, the first 12 trials were attention-fixed, while the remaining four were attention-switching (Figure 2a). The direction to which participants should attend was decided randomly for each test trial, with an equal number of left- and right-attention trials (i.e., eight trials for each side). As output, each type of model produced four detection results (corresponding to filters with four widths) and eight correlation coefficients (four for the target speech including filtered samples and four for the ignored speech) at each snippet. The results of online AAD models in Section 3.2.2. were reported using the data obtained during the test.

#### 2.3.4. Data Acquisition and Analysis

EEG data were recorded during the experiment using a 64-electrode system with Neuroscan SynAmps RT (64-channels Quik-Cap, Compumedics, Victoria, Australia) and CURRY 8 X recording software (Compumedics, Victoria, Australia). Raw EEG data were sampled at a rate of 1000 Hz and referenced to the voltage from an electrode positioned between the electrode sites Cz and CPz. Two VEOGs were installed above and below the left eye, respectively, and a pair of HEOG was attached to the lateral canthi of both eyes. The impedance of all electrodes was less than 10 kΩ. The EEG data were not directly utilized to analyze the online AAD results except for reproducing the results obtained during the experiment.

Figure 2b shows construction and testing of the online AAD model during the experiment. While recording EEG, data were transmitted to MATLAB (v9.5.0 R2018b, The MathWorks Inc., Natick, MA, USA) in the same computer in blocks of M second (s), corresponding to hopping size, via the streaming function of CURRY 8 X. For each trial, EEG data were accumulated in the pre-specified buffer in MATLAB after the onset of speech stimuli.

Speech envelopes were preprocessed from the speech stimuli and stored in MATLAB in advance. The preprocessing of these speech stimuli was performed as explained in Section 2.2.1 In contrast, the EEG data in the buffer were preprocessed in situ. Minimal preprocessing was conducted to reduce computational cost. After the EEG data were copied, they were re-referenced by a common average reference excluding V/HEOGs. Then, the component of the 2–8 Hz frequency band was extracted by a finite impulse response band-pass filter of the 1650th order. The filtered data were downsampled to 64 Hz and z-scored.

All of these procedures were implemented on a computer equipped with a central processing unit (CPU, i7-6700, Intel (R) Core (TM)) whose clock frequency was 3.40 GHz and had a random access memory (RAM) of 32 GB memory capacity. For construction and testing of the online decoder model, functions in mTRF toolbox (v 2.0) were used [28].

## 3. Results

### 3.1. Results of Online AAD Simulation

#### 3.1.1. Parameter Selection

**Figure 3:**
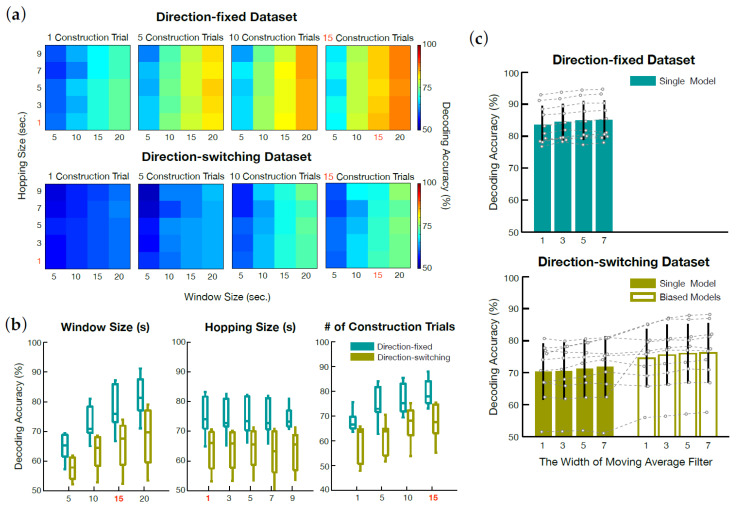
Online AAD simulation results. (**a**) Heatmaps showing an overview of changes in average detection accuracy according to parameter values for two datasets. Selected parameter values for the online AAD model are colored red. (**b**) Boxplots showing the effects of each parameter on detection accuracy. For each boxplot, the detection accuracies presented in (**a**) are collapsed to each parameter. The edges of each box denote the 25th and 75th quantiles, and the middle line in each box refers to the median. Again, the selected value of each parameter is colored red. (**c**) Online AAD simulation results from both datasets applying the selected parameters. A black line on each bar denotes ± 1 standard deviation.

Figure 3a shows an overview of the simulation results for parameter selection. As seen in the heatmap, the detection accuracy increased as W and I increased. This tendency was consistent across datasets. However, M did not seem to affect detection accuracy. We inspected the change of detection accuracy for each parameter in detail by collapsing the other two parameters in the heatmap. As shown in the boxplot in Figure 3b, a clear trend of increase of detection accuracy was seen in both datasets as W and I increased. In contrast, detection accuracy seemed to be independent of M.

Based on this result, we selected the values of parameters for the online AAD experiment. The hopping size M and the number of trials used for model construction I were chosen as 1 s and 15 trials, respectively, while the window size W was set as 15 s. The reason for choosing the value of window size W as 15 s, albeit the accuracies from the simulation were higher when the window size was 20 s, was to reduce the time it took for the model to produce the first detection result in each trial.

In addition, the direction-switching dataset collected in an environment more similar to that of the online AAD experiment showed no large difference in detection accuracy when *W* was 15 (71.41 ± 8.07%) or 20 (73.82 ± 8.07%) s and the other two parameters were set as above.

#### 3.1.2. Direction-Biased Model

The simulation results to investigate whether biased models outperform a single AAD model are presented in Figure 3c. This simulation was conducted separately from that in the previous section due to adjustment of the number of trials used in model construction for balancing the left- and right trials. Accordingly, the I determined in the previous section was adjusted to an even number of 14, and test trials were increased by one to a total of 16 trials. There was no result from the biased decoders with the direction-fixed dataset due to the inapplicability of biased decoders with target speech presented on only one side.

While the average detection accuracy of a single model built on the direction-switching datasets was 70.37 ± 8.77%, that with biased decoders was 74.54 ± 9.11%. Across individuals, detection accuracies of the biased models were higher than those of the single model in eight of 10 cases.

#### 3.1.3. Smoothing of Correlation Coefficients

Figure 3c also shows the smoothing effect on correlation coefficients for the direction-fixed dataset. The average detection accuracy without filters was 83.59 ± 5.84%, while that with a moving average filter (width: 7 samples) improved to 85.08 ± 6.15%. The same effect was seen with the direction-switching dataset. The average detection accuracy of a single model increased by about 1.6% after applying the moving average filter (71.97 ± 9.60%, width: 7 samples). For biased models, the average detection accuracy increased by about 1.67% after using the same filter (76.21 ± 9.35%). 

Even when the biased decoders were used with the moving average filter with 7-sample width, the detection accuracy in the direction-switching dataset was about 10% lower than that in the direction-fixed dataset without filtering. This difference was statistically significant (t(18)=2.51, p< 0.05).

### 3.2. Results of the Online AAD Experiment

#### 3.2.1. Behavioral Results

Participants successfully attended to the target speech and ignored the other speech throughout the experiment (Table 1), correctly answering 93.33 ± 4.08% of the questions about the target speech. In contrast, they correctly replied to only 33.06 ± 6.44% of the questions about the ignored speech, lower than the chance level (35%) determined by the binomial test at the significance level of 5% (n=60, p=0.25).

Results were similar when tested separately with construction trials and the test trials. In the model construction trials, participants correctly answered 93.45 ± 4.16% of the questions about the target speech, but only 36.90 ± 8.35% of the questions about the ignored speech, which was lower than the chance level (39.29%) determined by the binomial test at the significance level of 5% (n=28, p=0.25). In the test trials, the average correct answer rate of the questions about the target speech was 93.22 ± 5.01%, while that of the ignored speech was 29.69 ± 5.85%, which was also lower than the chance level (37.5%). There was no difference in average correct answer rate of the questions about target speech between model construction trials and test trials (t(5)=0.12, p> 0.05).

#### 3.2.2. Auditory Attention Detection Results

**Figure 4:**
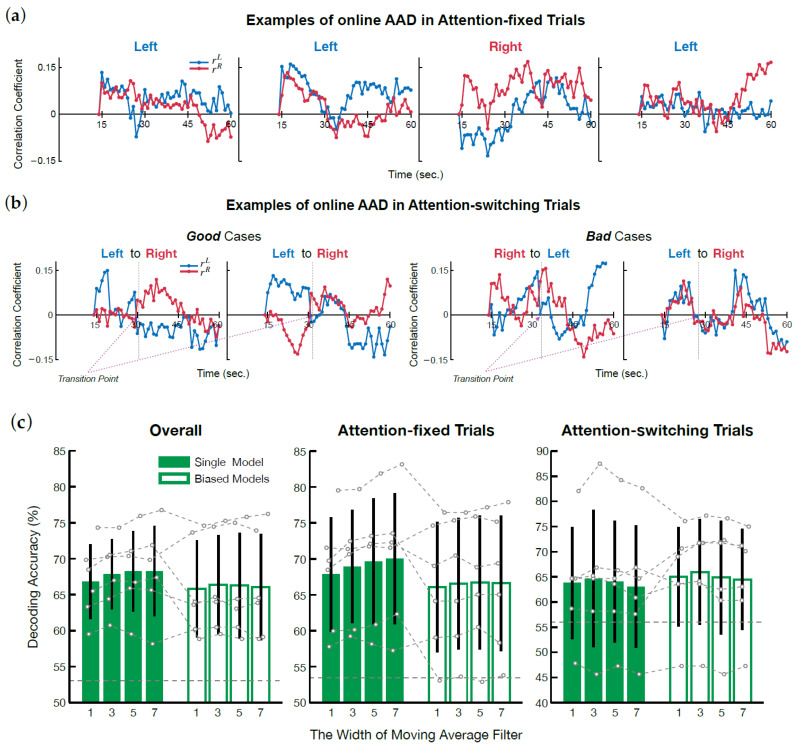
Online AAD experiment results. (**a**,**b**) Online AAD results of four attention-fixed trials and four attention-switching trials. (**c**) The average detection accuracy of all the participants for the 16 test trials, the attention-fixed trials (12 trials), and the attention-switching trials (4 trials). A black line on each bar denotes ± 1 standard deviation. A dashed gray horizontal line on the bottom of each plot signifies the chance level.

Figure 4a,b shows the changes of correlation coefficients between the envelope of the reconstructed speech and that of original speech. Except for the trial illustrated in the rightmost corner of Figure 4a, the model was able to track the direction of participants’ attention quite well.

In the examples plotted in Figure 4b, two ‘good’ and ‘bad’ cases for attention-switching trials are shown. Here, the direction of attention changed halfway through each trial. The good cases show that the model was able to track the sudden change in direction of attention quite well. However, there were some trials with undesirable results.

Figure 4c shows the average and individual detection accuracy of both the single model and biased models, including correlation coefficient smoothing, for test trials, attention-fixed trials, and attention-switching trials. For all 16 trials used in the test, the average detection accuracy of the single online AAD model was 66.82 ± 5.21%, and there was no participant with detection accuracy lower than the chance level (52.99%, n=736, p=0.5). When the moving average filter was applied, the average detection accuracy improved up to 68.27 ± 6.29%. In contrast, unlike our expectation, the average detection accuracy of the biased models (65.81 ± 6.74%, without filtering) was lower than that of the single model, and smoothing was not as effective.

For the attention-fixes trials (12 trials), the average detection accuracy of the single model was 67.84 ± 7.97% and improved to 70.02 ± 9.11% after applying the moving average filter (width: 7 samples). In the biased models, the detection accuracy tended to be low compared to the result from the single model (w/o filter: 66.06 ± 9.07%, w/ the filter: 66.61 ± 9.42%). Negligible effect was seen for smoothed correlation coefficients in the biased models. Nevertheless, detection accuracy of all individuals in all cases exceeded the chance level (53.44%, n=552, p=0.5).

For the attention-switching trials (4 trials), the average detection accuracy was slightly lower compared to that of the attention-fixed trials. When no filter was applied, the average detection accuracy of the single model was 63.77 ± 11.11%, while that of the biased models was 65.04 ± 9.89%. However, unlike the attention-fixed trials, the biased models produced a positive effect. The average detection accuracy of the biased model was about 1.2% higher than that of the single model, though the increase was not as great as with the direction-fixed dataset. When the smoothing filter was applied, the average of detection accuracy increased only when the width of the filter was 3 samples (the single model: 64.67 ± 13.64%, the biased models: 65.94 ± 9.89%). If the width of the filter was greater than 3 samples, it had no effect or adversely affected detection accuracy (with filter width of 7 samples, the single model: 63.04 ± 12.14%, the biased models: 64.49 ± 10.03%).

## 4. Discussion

In the present study, we proposed an online AAD model by introducing an overlapping sliding window to the linear decoder model and demonstrated model accuracy through simulation and online experiment. The online AAD model was constructed and implemented during a dichotomous listening task based on the streamed EEG data and produced a detection result every second. The detection accuracy of the model was promising, even though it was not on par with the simulation results based on two datasets acquired from our previous study. As additional ways of improving the detection accuracy of our model, we introduced biased models and correlation coefficient filtering. The positive effect of filtering was noted in both simulation and experiment. However, the biased models were beneficial only during the simulation and the attention-switching trials.

Few studies have conducted research on online AAD. Miran et al. suggested an AAD model based on a state-space model with Bayesian filtering that detected the direction of attention within a delay of 2 s in a competing-talker scenario (about 80% detection accuracy for three participants) [23]. However, the model was applied only to previously recorded EEG data, which did not replicate real-world applications. In addition, two studies have attempted to implement online AAD models in a specific domain. Haghighi et al. implemented AAD while adjusting the volume of both attended and ignored speech by applying the detection output in probability as weights for the gain control [6]. Zink et al. conducted feedback training by presenting the correlates of the attentional state of the listeners, which were produced by their AAD model [8]. Their models showed about less than 80% and 79.7 ± 7.0% detection accuracies on average, respectively, both of which were higher than that found in the present study except for the simulation result from the direction-fixed dataset. Haghighi et al. and Zink et al. used larger amounts of data to construct their online models (30 and 24 min., respectively) and consistently provided feedback to participants throughout the test time to promote attention to the target sound.

However, our online AAD model had a better time resolution (on the order of 1 s), compared to that of the models proposed by Haghighi et al. and Zink et al. (on the order of 20 s and 10 s, respectively) and showed a possibility to capture attention transition within a few seconds. Even taking into account the possible effect of the visual cues presented prior to the transition point on the sensitivity, our model reacted faster than that suggested by O’Sullivan et al. despite the same window size [26]. The comparative features of the prior online AAD studies and the present study are presented in Table 2.

From the simulation and experiment, we found that detection accuracy differs by experimental condition. The average detection accuracy of the direction-fixed dataset was about at least 10% higher than those from the direction-switching dataset and the experiment. This indicates that detection of changed direction of attention during a task is challenging. According to recent studies, auditory attention switching requires an increase of listening effort [29], which can affect neurophysiological responses and brain regions involved in listening [30,31,32,33]. These changes could influence AAD results by affecting the neural representation of speech. In line with this idea, detection performance in the attention-switching trials was lower than those in other experimental conditions (i.e., attending to a single side throughout the task or changing the direction of attention across trials), implying that it is more difficult for participants to keep track of target speech when the direction of attention changes.

As previous studies have found a spatial effect on AAD [9,24], we constructed direction-biased models and tested whether they showed better performance compared to a single model. The spatial effect on AAD was identified during simulation with the direction-switching dataset; this result was not reproduced during the experiment. The reason for these inconsistent results is not clear. Further studies are needed to verify the spatial effect on AAD and whether construction of an online decoder model based on this spatial effect is more effective in detecting the direction of auditory attention.

The proposed model has various potential applications. For example, with adaptive hearing aids, output of the model such as detection results or correlation coefficients could be used to control the gain of each speech sound [6]. Moreover, the model can be used to develop a protocol of feedback training to improve auditory attention. Studies on neurofeedback training have suggested that neural activity can be influenced by providing the correlates of neuronal activity as feedback, resulting in changes in related cognitive processes [8,34,35,36]. Speech-in-noise perception could be improved with this feedback training, considering a recent report that the degree of neural representation of speech could predict speech intelligibility [37].

For the proposed model to be used for these applications, several improvements are needed. The average detection accuracy of our model was about 70%, which is not a satisfactory level. Extending the frequency band of EEG for the model can be considered as a possible way to improve the detection accuracy. Recently, many studies have argued that neural signals below 2 Hz may reflect representation of prosodic information or larger linguistics unit such as phrases [38,39,40]. This information could be beneficial for the model to identify a participant′s auditory attention because it would give additional clues to their state, in terms of speech processing. Moreover, the model performance would increase if an online AAD model was constructed only on data acquired when participants genuinely paid attention to the target speech, excluding snippets where participants did not attend to the target speech properly. To do this, a method should be devised to identify where or to what speech the participant is attending rather than relying on comprehension questions. Plus, the linear model used in this study can be replaced with a non-linear model. Since such a model is usually computationally more expensive than a linear model, research on reduction of computational cost should be performed. Lastly, the variability of individual detection accuracy was quite large. To ensure that the proposed model works well for every individual, further research is required to inspect determinant factors contributing to such variability and reduce their effects.

## 5. Conclusions

In this study, we proposed an online AAD model by introducing an overlapping sliding window to the linear decoder model and demonstrated the model accuracy through a simulation and an online experiment using EEG. The proposed model achieved the detection accuracy of up to about 85% in the simulation and up to about 70% in the online experiment, which demonstrated the feasibility of the model application, and operated in high temporal resolution.

## Figures and Tables

**Table 1:** The average correct answer rate (%) and standard deviation across six participants.

Question	Overall Trials	Model Construction Trials	Test Trials
Target	93.33 (4.08)	93.45 (4.16)	93.22 (5.01)
Ignored	33.06 (6.44)	36.90 (8.35)	29.69 (5.85)

**Table 2:** Comparisons of online auditory attention detection (AAD) model implementation.

Comparative Features	Zink et al. [8]	Haghighi et al. [6]	Present Study
Method	Linear Decoder Model	Regularized Discriminant Analysis	Linear Decoder Model w/ an Overlapping Sliding Window
Number of Participants	12	10	6
Training Time (min.)	24	30	14
Time Resolution (s)	10	20	1
Feedback	O	O	X
Transient Switching of Attention	X	X	O
Voice	male	male + female	male
Stimuli Presentation	dichotic	diotic	dichotic
Frequency Band of EEG (Hz)	1–8 Hz	1.5–10 Hz	2–8 Hz
Average Accuracy (%)	79.7	Less than 80	85.08 * 67.84~71.97 ^†^ 64.67 ^‡^

* Direction-fixed dataset. ^†^ Direction-switching dataset and attention-fixed trials in the online experiment. ^‡^ Attention-switching trials in the online experiment.

## Data Availability

The data presented in this study are available on request from the corresponding authors. The data are not publicly available due to privacy.

## Abbreviations

Auditory attention detection, AAD; electroencephalogram/electroencephalography, EEG; event-related potentials, ERP; root square mean, RMS; vertical electrooculogram/electrooculography, VEOG; horizontal electrooculogram/electrooculography, HEOG; central processing unit, CPU; random access memory, RAM.
